# Weight-related concerns and weight-control behaviors among overweight adolescents in Delhi, India: A cross-sectional study

**DOI:** 10.1186/1479-5868-8-9

**Published:** 2011-02-07

**Authors:** Melissa H Stigler, Monika Arora, Poonam Dhavan, Radhika Shrivastav, K Srinath Reddy, Cheryl L Perry

**Affiliations:** 1 Michael & Susan Dell Center for Healthy Living, School of Public Health, University of Texas; Austin and Houston, Texas, USA; 2 HRIDAY (Health-Related Information Dissemination Amongst Youth); Delhi, India; 3 Public Health Foundation of India (PHFI); Delhi, India

## Abstract

**Background:**

Obesity is emerging as a public health problem among adolescents in India. The aim of this study was to describe specific weight-related concerns among school-going youth in Delhi, India and to assess the prevalence of weight control behaviors, including healthy and unhealthy ones. Differences by weight status, gender, grade level, and school-type (a proxy for SES in this setting) are considered.

**Methods:**

This study is cross-sectional by design. A sample of eighth and tenth graders (n = 1818) enrolled in Private (middle-high SES) and Government (low SES) schools (n = 8) in Delhi, India participated. All students' height and weight were measured. Students participated in a survey of weight-related concerns and weight-control behaviors, as well. Mixed-effects regression models were used to test for differences in weight-related concerns and weight-control behaviors across key factors of interest (i.e., weight status, gender, grade level, and SES).

**Results:**

The combined prevalence of obesity and overweight was 16.6%, overall. Controlling one's weight was important to overweight and non-overweight youth, alike (94.2% v. 84.8%, *p *< 0.001). Significantly more overweight or obese youth reported trying to control their weight last year, compared to those who were not overweight (68.1% v. 18.0%, *p *< 0.001). Healthy weight control behaviors were more common than unhealthy or extreme practices, although the latter were still prevalent. Half of the overweight or obese students misclassified their weight status, while about 1 in 10 non-overweight youth did the same. Body dissatisfaction was highest among overweight youth and girls.

**Conclusions:**

Interventions to promote healthy weight control should be pertinent to and well-received by school-going youth in India. Healthy weight control practices need to be explicitly encouraged and unhealthy practices reduced. Future interventions should address issues specific to body image, too, as body dissatisfaction was not uncommon among youth.

## Findings

Obesity is emerging as a serious public health problem in India, especially in urban regions of the country [1,2]. Here, obesity may ultimately prove to be more problematic than that being experienced in the United Kingdom and the United States, as its negative health sequelae, like diabetes, occur at least a decade earlier in the life course, by comparison [3]. At present, the prevalence of obesity among affluent adolescents in India is comparable to that among adolescents in many industrialized countries in the West [2,4-7]. There is a clear need for weight control interventions here, and young people should be an important target [2]. To date, no published study has evaluated the strategies adolescents use in India to control their weight. This paper reports on specific weight-related concerns and the prevalence of weight control behaviors among adolescents (13-17 years old) in Delhi, India. Differences by weight status, gender, grade level, and school type (which is a proxy for SES in India [8]) are explored.

This study is cross-sectional by design. In 2006, eight schools in Delhi, India were recruited to participate. Half (n = 4) were Private schools (which cater to middle-upper SES families) and half (n = 4) were Government schools (which cater to low SES families). Ethical clearances for the study were obtained from the appropriate human subjects boards in India and the US. All students enrolled in the 8^th ^and 10^th ^grades in these eight schools (n = 2339) were eligible and invited to participate in a survey and have their height and weight measured. Response rates for anthropometric measures and survey were 87.2% and 88.6%, respectively. Non-participants included parent refusals (<1%), student refusals (<1%), and student absentees (7-11%). The analysis sample includes 1,818 students who participated in both data collection efforts. Of these, 60% were boys, 52% were in Private schools, and 55% were in 8^th ^grade. The mean age of these 8^th ^and 10^th ^graders was 13.9 years old and 15.8 years old, respectively.

Measured height and weight were collected from these students using standardized protocols adapted from Lohman [9]. These measures were used to compute each student's BMI (kg/m^2^), then their weight status was derived using new age- and gender-specific BMI cut-points provided by WHO [10]. This international standard is especially appropriate for school-going youth in India [6]. Measures of weight-related concerns and weight control behaviors were adapted from an instrument previously used in the United States [11]. Weight-related concerns included (a) perceived weight status, (b) body satisfaction, and (c) the importance of weight control. Weight control practices included both (a) healthy and (b) unhealthy behaviors. Surveys were administered in Hindi and English based on the medium of instruction in school. Further details about these measures, including item wording, are provided in Table footnotes.

Mixed-effects regression models were used to test for differences in weight-related concerns and weight control behaviors across factors of interest (e.g., weight status, gender, grade level, and school type). This type of regression is appropriate for study designs like this, where students are sampled within schools [12]. School was specified as a random effect. Differences in these variables were examined by weight status, for all students (Table 1) and within demographic sub-groups (Table 2). For the latter set of analyses, odds ratios and 95% confidence intervals were generated using logistic regression models, using underweight and normal weight students as the referent group. Finally, differences in weight-related concerns and weight control behaviors were examined by gender and school type, for overweight or obese adolescents only (Table 3). All analyses were conducted using *xtmelogit *in STATA v10. No differences in grade level for the analyses were noted, so these results are not reported.

**Table 1:** Differences in weight-related concerns and weight-control behaviors, by weight status; Delhi, India; 2006 (n = 1818).

	**Overweight or obese**^**1 **^**(n = 300)**		**Underweight or normal weight**^**1 **^**(n = 1518)**		
	**Percent**	**(95% CI)**	**Percent**	**(95% CI)**	**p-value**^**2**^

***WEIGHT-RELATED CONCERNS***					

**Importance of weight control**^**3**^					

Important	94.2	(90.2 - 96.6)	84.8	(79.8 - 88.7)	<0.001

Not important	5.8	(3.4 - 9.8)	15.2	(11.3 - 20.2)	<0.001

**Perceived weight status**^**4**^					

Overweight	56.2	(47.8 - 64.4)	10.8	(8.4 - 13.7)	<0.001

About the right weight	25.0	(20.4 - 30.3)	49.3	(46.8 - 51.9)	<0.001

Underweight	16.4	(11.9 - 22.1)	39.4	(34.7 - 44.2)	<0.001

**Perceived body satisfaction**^**5**^					

Low	55.1	(49.3 - 60.7)	26.7	(24.5 - 29.0)	<0.001

Moderate	22.8	(17.9 - 28.5)	36.8	(33.6 - 40.1)	<0.001

High	23.1	(17.6 - 29.7)	36.2	(31.7 - 40.9)	<0.001

***WEIGHT-CONTROL BEHAVIORS***					

Tried to lose weight in the last year ^6^	71.9	(66.1 - 77.1)	34.5	(31.8 - 37.3)	<0.001

**Healthy behaviors (at least one)**^**7**^	91.5	(87.5 - 94.4)	82.9	(79.9 - 85.5)	<0.001

Exercised more	76.7	(71.0 - 81.7)	55.2	(52.1 - 58.3)	<0.001

Ate less sweets	69.2	(62.7 - 75.1)	49.1	(45.1 - 53.2)	<0.001

Ate less high fat foods	63.2	(57.3 - 68.7)	42.1	(39.3 - 44.9)	<0.001

Ate more fruits and vegetables	65.2	(57.9 - 72.0)	64.3	(59.4 - 68.9)	0.772

**Unhealthy behaviors (at least one)**^**7**^	77.7	(69.8 - 84.0)	62.3	(54.5 - 69.6)	<0.001

Fasted	38.0	(28.2 - 48.9)	34.0	(26.1 - 42.8)	0.243

Skipped meals	39.0	(29.0 - 50.0)	27.8	(20.8 - 36.0)	0.001

Ate very little food	55.5	(47.5 - 63.2)	28.9	(24.3 - 33.9)	<0.001

Used food substitute	11.6	(7.5 - 17.5)	13.4	(9.7 - 18.1)	0.392

Took diet pills	15.1	(10.0 - 22.1)	10.7	(7.6 - 14.7)	0.035

Made myself vomit	8.3	(4.8 - 14.3)	6.9	(4.4 - 10.7)	0.357

^1 ^Weight status determined using age- and gender-specific cut-points from WHO [10].

^2 ^Test for differences performed using mixed-effects regression models, with school specified as a random effect [12]. Models were unadjusted.

^3 ^Importance of weight control was measured with one item, "How important is controlling your weight (a) very important, (b) important, (c) not important, (d) not at all important?" The responses were collapsed into two categories: (1) important ("very important," "important") and (2) not important ("not important," "not at all important") [11].

^4 ^Perceived weight status was measured by a single question, "At this time, do you feel that you are (a) very underweight, (b) somewhat underweight, (c) about the right weight, (d) somewhat overweight, or (e) very overweight?" The responses were collapsed to create three categories: (1) underweight ("very underweight" and "somewhat underweight"), (2) the right weight ("about the right weight"), and (3) overweight ("somewhat overweight" and "very overweight") [11].

^5 ^Perceived body satisfaction was measured using a modified version of the Body Satisfaction Scale, which included five items assessing one's satisfaction with different parts of their body (i.e., height, weight, body shape, waist, hips) using a Likert scale that ranged from "not at all satisfied" to "very satisfied." Responses to these questions were summed and categorized as low, moderate, and high based on distributions within this study population, with one third of the population in each category [11].

^6 ^Students who responded "yes" to "In the past year, have you done anything to try to lose weight or avoid gaining weight ... (a) yes, (b) no?" [11].

^7 ^Students who responded "yes" to "During the past one year, have you done any of the following things in order to lose weight or avoid gaining weight ... [behaviors] ... (a) yes, (b) no?" [11].

**Table 2:** Differences in weight-related concerns and weight-control behaviors, by weight status ^1^, within demographic sub-groups; Delhi, India; 2006 (n = 1818).

	All students (n = 1818)							
	**Girls (n = 722)**		**Boys (n = 1096)**		**Private (n = 949)**		**Government (n = 869)**	

	**OR**^**2**^	**(95% CI)**	**OR**^**2**^	**(95%CI)**	**OR**^**2**^	**(95% CI)**	**OR**^**2**^	**(95%CI)**

***WEIGHT-RELATED CONCERNS***								

**Importance of weight control**^**3**^								

Important	3.18	(1.23 - 8.23)	2.92	(1.66 - 5.15)	3.32	(2.01 - 5.49)	1.73	(0.41 - 7.41)

**Perceived weight status**^**4**^								

Overweight	10.16	(6.16 - 16.76)	12.73	(8.62 - 18.80)	10.52	(7.49 - 14.76)	8.23	(3.98 - 16.99)

**Body satisfaction**^**5**^								

Low	4.03	(2.52 - 6.42)	3.05	(2.20 - 4.23)	3.40	(2.51 - 4.59)	2.41	(1.22 - 4.77)

***WEIGHT-CONTROL BEHAVIORS***								

Tried to lose weight in the last year ^6^	5.20	(3.10 - 8.71	4.55	(3.19 - 6.50)	4.68	(3.39 - 6.44)	5.38	(2.48 - 11.67)

**Healthy behaviors (at least one)**^**7**^	5.24	(1.89 - 14.55)	1.75	(1.07 - 2.87)	2.06	(1.31 - 3.24)	6.46	(0.87 - 47.66)

Exercised more	4.20	(2.35 - 7.50)	2.03	(1.42 - 2.90)	2.23	(1.61 - 3.09)	4.46	(1.83 - 10.87)

Ate less sweets	4.27	(2.46 - 7.40)	1.75	(1.24 - 2.47)	2.36	(1.75 - 3.19)	3.24	(1.39 - 7.55)

Ate less high fat foods	3.04	(1.94 - 4.77)	2.17	(1.54 - 3.06)	2.50	(1.85 - 3.37)	1.88	(0.93 - 3.79)

Ate more fruits and vegetables	1.79	(1.11 - 2.87)	0.83	(0.59 - 1.18)	1.04	(0.76 - 1.42)	0.82	(0.40 - 1.66)

**Unhealthy behaviors (at least one)**^**7**^	4.09	(2.30 - 7.27)	1.51	(1.07 - 2.15)	2.23	(1.63 - 3.05)	1.70	(0.73 - 3.99)

Fasted	1.78	(1.10 - 2.88)	0.90	(0.62 - 1.31)	1.23	(0.89 - 1.69)	1.18	(0.57 - 2.46)

Skipped meals	2.05	(1.26 - 3.35)	1.45	(1.00 - 2.10)	2.19	(1.58 - 3.04)	0.58	(0.27 - 1.26)

Ate very little food	4.37	(2.73 - 7.00)	2.56	(1.82 - 3.60)	2.82	(2.08 - 3.82)	6.12	(2.81 - 13.34)

Used food substitute	1.49	(0.81 - 2.76)	0.64	(0.40 - 1.03)	0.81	(0.55 - 1.19)	0.95	(0.28 - 3.17)

Took diet pills	2.26	(1.16 - 4.39)	1.20	(0.76 - 1.87)	1.38	(0.93 - 2.06)	1.92	(0.76 - 4.82)

Made myself vomit	1.48	(0.64 - 3.38)	1.08	(0.64 - 1.82)	1.07	(0.66 - 1.72)	2.43	(0.90 - 6.57)

^1 ^Weight status determined using age- and gender-specific cut-points from WHO [10].

^2 ^Test for differences performed using mixed-effects regression models, with school specified as a random effect [12]. Models were unadjusted.

^3 ^Importance of weight control was measured with one item, "How important is controlling your weight (a) very important, (b) important, (c) not important, (d) not at all important?" The responses were collapsed into two categories: (1) important ("very important," "important") and (2) not important ("not important," "not at all important") [11].

^4 ^Perceived weight status was measured by a single question, "At this time, do you feel that you are (a) very underweight, (b) somewhat underweight, (c) about the right weight, (d) somewhat overweight, or (e) very overweight?" The responses were collapsed to create three categories: (1) underweight ("very underweight" and "somewhat underweight"), (2) the right weight ("about the right weight"), and (3) overweight ("somewhat overweight" and "very overweight") [11].

^5 ^Perceived body satisfaction was measured using a modified version of the Body Satisfaction Scale, which included five items assessing one's satisfaction with different parts of their body (i.e., height, weight, body shape, waist, hips) using a Likert scale that ranged from "not at all satisfied" to "very satisfied." Responses to these questions were summed and categorized as low, moderate, and high based on distributions within this study population, with one third of the population in each category [11].

^6 ^Students who responded "yes" to "In the past year, have you done anything to try to lose weight or avoid gaining weight ... (a) yes, (b) no?" [11].

^7 ^Students who responded "yes" to "During the past one year, have you done any of the following things in order to lose weight or avoid gaining weight ... [behaviors] ... (a) yes, (b) no?" [11].

**Table 3:** Differences in weight-related concerns and weight-control behaviors, by gender and school type; Delhi, India; 2006 (n = 300 overweight or obese students only).

	**Overweight or obese (n = 300)**^**1**^									
	**Girls (n = 107)**		**Boys (n = 193)**			**Private (n = 265)**		**Government (n = 35)**		

	**Percent**	**(95% CI)**	**Percent**	**(95%CI)**	**p-value**^**2**^	**Percent**	**(95% CI)**	**Percent**	**(95%CI)**	**p-value**^**2**^

***Weight-related concerns***										

**Importance of weight control**^**3**^										

Important	95.5	(88.5 - 98.3)	91.6	(84.7 - 95.5)	0.212	92.8	(87.0 - 96.1)	94.0	(78.6 - 98.5)	0.804

Not important	4.5	(1.7 - 11.5)	8.4	(4.5 - 15.3)	0.212	7.2	(3.9 - 13.0)	6.0	(1.5 - 21.5)	0.804

**Perceived weight status**^**4**^										

Overweight	66.7	(53.2 - 77.9)	54.2	(42.1 - 65.8)	0.047	64.4	(53.7 - 73.9)	43.3	(25.9 - 62.0)	0.054

About the right weight	17.1	(11.1 - 25.6)	29.4	(23.3 - 36.3)	0.022	24.1	(19.3 - 29.7)	31.4	(18.3 - 48.3)	0.351

Underweight	13.9	(5.8 - 29.6)	12.2	(5.4 - 25.4)	0.690	8.8	(3.6 - 20.0)	23.8	(9.2 - 49.2)	0.113

**Body satisfaction**^**5**^										

Low	62.6	(51.0 - 72.9)	50.4	(40.9 - 59.9)	0.052	57.4	(48.1 - 66.1)	45.1	(28.4 - 62.9)	0.237

Moderate	23.5	(16.3 - 32.7)	23.8	(18.2 - 30.4)	0.961	25.4	(20.4 - 31.1)	11.4	(4.4 - 26.8)	0.078

High	16.2	(7.7 - 31.0)	28.7	(16.6 - 44.9)	0.036	16.3	(9.8 - 25.8)	43.2	(25.0 - 63.4)	0.008

***Weight-control behaviors***										

Tried to lose weight in the last year ^6^	78.4	(68.5 - 85.9)	68.7	(61.1 - 77.1)	0.110	72.7	(64.8 - 79.4	73.7	(55.6 - 86.2)	0.913

**Healthy behaviors (at least one)**^**7**^	97.2	(90.8 - 99.2)	90.7	(80.8 - 95.7)	0.024	91.3	(84.1 - 95.4)	97.3	(82.2 - 99.6)	0.258

Exercised more	85.3	(75.5 - 91.7)	74.3	(65.4 - 81.6)	0.031	76.8	(71.3 - 81.6)	82.4	(65.9 - 91.9)	0.471

Ate less sweets	82.3	(72.0 - 89.3)	60.3	(50.6 - 69.3)	<0.001	65.5	(59.5 - 71.1)	78.8	(61.7 - 89.5)	0.132

Ate less high fat foods	70.2	(60.6 - 78.4)	59.1	(51.6 - 66.3)	0.060	63.9	(56.9 - 70.3)	58.8	(41.7 - 74.0)	0.577

Ate more fruits and vegetables	75.8	(64.7 - 84.2)	61.9	(51.9 - 71.1)	0.018	69.2	(59.0 - 77.9)	58.5	(39.5 - 75.3)	0.306

**Unhealthy behaviors (at least one)**^**7**^	84.2	(75.9 - 89.9)	67.4	(60.4 - 73.6)	0.002	72.5	(66.8 - 77.5)	80.0	(63.6 - 90.2)	0.346

Fasted	43.6	(26.6 - 62.2)	30.3	(17.7 - 46.7)	0.037	32.2	(15.6 - 55.0)	39.6	(14.5 - 71.7)	0.703

Skipped meals	39.6	(30.6 - 49.4)	32.4	(26.1 - 39.5)	0.224	36.1	(30.4 - 42.2)	26.5	(14.4 - 43.5)	0.273

Ate very little food	64.4	(54.8 - 73.0)	49.5	(42.3 - 56.6)	0.015	52.4	(46.2 - 58.4)	73.5	(56.5 - 85.6)	0.023

Used food substitute	17.5	(11.3 - 26.0)	15.0	(10.5 - 20.8)	0.577	16.8	(12.7 - 21.9)	8.8	(2.9 - 24.0)	0.241

Took diet pills	15.4	(9.5 - 24.2)	19.7	(14.2 - 26.6)	0.371	18.3	(13.6 - 24.1)	17.4	(7.8 - 34.5)	0.908

Made myself vomit	7.1	(2.7 - 17.4)	11.4	(5.26 - 23.1)	0.216	9.9	(4.3 - 21.1)	9.9	(2.2 - 34.7)	0.998

^1 ^Weight status determined using age- and gender-specific cut-points from WHO [10].

^2 ^Test for differences performed using mixed-effects regression models, with school specified as a random effect [12]. Models were unadjusted.

^3 ^Importance of weight control was measured with one item, "How important is controlling your weight (a) very important, (b) important, (c) not important, (d) not at all important?" The responses were collapsed into two categories: (1) important ("very important," "important") and (2) not important ("not important," "not at all important") [11].

^4 ^Perceived weight status was measured by a single question, "At this time, do you feel that you are (a) very underweight, (b) somewhat underweight, (c) about the right weight, (d) somewhat overweight, or (e) very overweight?" The responses were collapsed to create three categories: (1) underweight ("very underweight" and "somewhat underweight"), (2) the right weight ("about the right weight"), and (3) overweight ("somewhat overweight" and "very overweight") [11].

^5 ^Perceived body satisfaction was measured using a modified version of the Body Satisfaction Scale, which included five items assessing one's satisfaction with different parts of their body (i.e., height, weight, body shape, waist, hips) using a Likert scale that ranged from "not at all satisfied" to "very satisfied." Responses to these questions were summed and categorized as low, moderate, and high based on distributions within this study population, with one third of the population in each category [11].

^6 ^Students who responded "yes" to "In the past year, have you done anything to try to lose weight or avoid gaining weight ... (a) yes, (b) no?" [11].

^7 ^Students who responded "yes" to "During the past one year, have you done any of the following things in order to lose weight or avoid gaining weight ... [behaviors] ... (a) yes, (b) no?" [11].

The combined prevalence of overweight and obesity in our sample was 16.6% (n = 300). The prevalence of underweight, by comparison, was 15.4% (n = 281). The combined prevalence of overweight and obesity was more than six times higher in Private schools (higher SES) than Government schools (lower SES) (26.6% vs. 4.0%, *p *< 0.001), consistent with prior research [3]. No significant differences in the combined prevalence were noted by grade level or gender [6].

Almost all of these youth, regardless of weight status, reported that controlling their weight was important to them (Table 1). This was especially true for overweight or obese students (Table 1), and, within this group, was true for girls and boys and those enrolled in Private and Government schools (Table 3). Moreover, almost three-quarters of overweight or obese youth reported trying to lose weight in the last year, while almost one-third of non-overweight youth reported the same (71.9% vs. 34.5%, *p *< 0.001; Table 1). Among overweight or obese youth, no differences in this effort to lose weight in the last year were observed by gender or school type (Table 3). Given these findings, interventions designed to help students manage their weight appropriately would be pertinent, then, to a reasonably large and diverse audience in India and so should be well received by the adolescents here. By comparison, the percentage of young people in this study that reported trying to lose weight in the last year is on par with other countries in South East Asia, like Japan [13], but exceeds that of adolescents in the US [14] and young adults in Western Europe, Eastern Europe, and South America [13].

Healthy and unhealthy weight control behaviors were common for overweight and non-overweight youth alike (Table 1). Among overweight or obese youth, almost all youth (91.5%) engaged in at least one healthy weight control behavior over the last year, while about three-quarters (77.7%) engaged in at least one unhealthy weight control behavior (Table 1). Among overweight or obese youth, girls were more likely than boys to engage in unhealthy (84.2% vs. 67.4%, *p *= 0.002; Table 3) behaviors, particularly, but no differences were noted by school type. Among the unhealthy behaviors, fasting, skipping meals, and eating very little food were the most common. Alleviating these behaviors, therefore, should be an important target of future interventions. Longitudinal research suggests these behaviors will lead to increased weight gain, not weight loss [15]. Skipping breakfast, in particular, can be especially problematic for overweight and non-overweight youth alike, leading to nutritional problems and interfering with learning and academic performance [16]. Skipping meals increases future risk for more extreme weight control behaviors, like the use of diet pills and self-induced vomiting, too [15].

The prevalence of these extreme dieting behaviors were either on par with (e.g., self-induced vomiting) or exceeded (e.g., taking dieting pills) prevalence rates previously reported in similar studies in the United States [17,18], Australia [19], and Israel [20]. This is alarming, as eating-related pathologies are beginning to emerge in India, too [21]. Half of the overweight or obese youth had low body satisfaction, while one-quarter of the non-overweight youth felt the same (Table 1). Among overweight or obese youth, girls were more likely than boys to perceive themselves as overweight (*p *= 0.047) and to have low body satisfaction (*p *= 0.052) (see Table 3). Concerns about excess weight and poor body satisfaction are more common among girls than boys, globally [13,14]. Future interventions developed to promote healthy weight control in India will, therefore, need to address issues specific to body image, too. In India, as elsewhere in the world, adolescents will need help negotiating the socio-cultural determinants of body dissatisfaction supported by the media, particularly the movie industry (i.e., "Bollywood") here.

Limitations of this study include its cross-sectional design and reliance on a relatively simple set of indicators for these weight-related concerns and behaviors. A more detailed assessment of these variables is not typically feasible for use in a large-scale epidemiologic study such as this, so we relied on past inventories [11,13,14]. We measured weight status, instead of relying on self-report, which strengthens our confidence in findings reported here.

Given the high prevalence of obesity among students in Private schools, these schools would be an appropriate setting for preventive interventions. Both boys and girls could benefit from preventive interventions, though these programs might be tailored to suit their specific needs (e.g., implementing girls-only or boys-only PE classes). Many overweight youth in this study were motivated to lose weight or avoid gaining weight and were taking action to do so. It was encouraging to document that healthy weight control practices were more prevalent than unhealthy or extreme ones. Many of these healthy weight control practices are recommended for adolescents and are often a behavioral target in obesity interventions [16,22,23]. Given the rapidity with which this obesity epidemic appears to be emerging, "translating" evidence-based interventions from the West for use in India may be an efficient way to begin to combat this problem [24]. More formative research is needed to ensure that these interventions with school-going youth in India are culturally-appropriate and shall adequately address the context in which these youth face and deal with weight-related concerns to maintain a healthy weight.

## Competing interests

The authors declare that they have no competing interests.

## Authors' contributions

MS led and conceptualized the study, designed the survey instruments, and drafted the manuscript. MA assisted in the conceptualization of the study, including the design of the survey. PD did the data analysis. RS led the data collection effort and assisted with data analysis. KSR and CP assisted in the conceptualization of the study, including the design of the survey. All authors read and approved the final manuscript.

## Authors' information

This team of researchers in the US (MS, CP, PD) and India (MA, RS, KSR) have been engaged in collaborative, NIH-funded research on health promotion among adolescents in India for the last 10 years. They are in the process of currently extended their work in tobacco control to focus on obesity, which is now another emerging problem and risk factor for chronic disease in India.
